# Advancements in Elastography for Evaluating Fibrosis in Renal Transplants: Current Perspectives

**DOI:** 10.3390/biomedicines12122671

**Published:** 2024-11-23

**Authors:** Giulio Distefano, Salvatore Granata, Walter Morale, Antonio Granata

**Affiliations:** 1 Institute of Nephrology and Dialysis, Maggiore Hospital of Modica, ASP Ragusa, 97015 Modica, Italy; 2 Department of Experimental and Clinical Medicine, University of Florence, 50121 Florence, Italy; 3 Unit of Urological Robotic Surgery and Renal Transplantation, University of Florence, Careggi Hospital, 50121 Florence, Italy; 4 Nephrology and Dialysis Unit, Cannizzaro Hospital, 95100 Catania, Italy

**Keywords:** renal transplant, ultrasound, renal ultrasound, elastography, shear wave velocity, renal stiffness

## Abstract

Renal fibrosis is a leading cause of chronic allograft nephropathy. While renal biopsy remains the gold standard for diagnosing fibrosis, it is an invasive procedure with potential for severe complications. Elastography, an emerging ultrasound imaging technique, appears to be a valuable tool for quantifying tissue stiffness, which correlates with fibrosis. Indeed, numerous studies have demonstrated a strong correlation between increased tissue stiffness, measured by elastography, and the degree of fibrosis detected in biopsy. Over the past few years, various elastography techniques have been evaluated, including strain elastography, shear wave elastography, and acoustic radiation force impulse. However, challenges such as operator dependence, tissue heterogeneity, and the lack of standardized protocols persist. Despite these limitations, elastography presents itself as a valuable tool for the non-invasive monitoring of renal transplant function and could facilitate the early detection of fibrosis, allowing for timely interventions. Future research should focus on standardizing acquisition protocols, establishing robust reference values, and exploring the clinical utility of elastography in guiding therapeutic decisions. The aim of this review is to explore the current state of elastography in the assessment of fibrosis in renal transplantation.

## 1. Introduction

Tubulointerstitial fibrosis with tubular atrophy represents the primary pathogenetic mechanism underlying chronic allograft nephropathy (CAN), regardless of the underlying cause. The continuous immune response, both cellular and humoral, exacerbated by the action of calcineurin inhibitors and various comorbidities (e.g., hypertension, diabetes, etc.) determines a persistent inflammatory state in the tubulointerstitial compartment, with loss of the vascular bed and replacement of normal tissue with fibrotic tissue [1,2]. Organ fibrosis thus appears to be the common endpoint of all mechanisms of chronic kidney damage; its extent is correlated with the rate of progression to end-stage renal disease (ESRD), and its quantification over time can provide useful information on therapeutic management [3]. Currently, the gold standard for quantifying fibrosis is the histological evaluation of a renal parenchyma biopsy specimen. While highly precise and reproducible, percutaneous renal biopsy is an invasive procedure associated with potential complications. A non-invasive method capable of adequate follow-up at low cost and with a high safety profile would be invaluable.

Ultrasonography has long been a cornerstone of renal graft monitoring, providing both morphological and functional information, including insights into the vascular system, a well-established clinical tool [4]. Among the various ultrasonographic techniques, the most relevant is elastography, which is based on the principle that the replacement of healthy tissue with fibrotic tissue alters the response to mechanical stimulation, increasing tissue stiffness [5]. Recently, numerous studies have demonstrated the potential role of elastography in the evaluation of graft fibrosis, but the data are not univocal. The aim of this review is to analyze the state of the art of the application of multiparametric imaging based on elastography in the study of the transplanted kidney and to discuss the possible future developments in this field.

## 2. Principles of Operation and Comparison of Techniques

**Figure 1:**
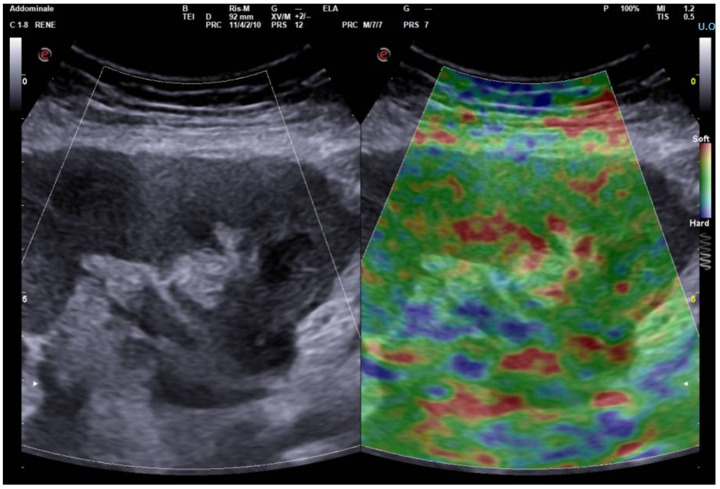
This figure presents a comparative visualization of a colorimetric elastographic map (**right**) and a traditional B-mode grayscale ultrasound image (**left**) of the kidney. The colorimetric map represents the spatial distribution of tissue stiffness, with different colors corresponding to varying degrees of elasticity. The reference scale provides a quantitative interpretation of the color-coded stiffness values. While this dual-modality approach offers a visual representation of tissue heterogeneity, it should be noted that the colorimetric map does not provide specific multiparametric information and therefore this visualization technique, when applied to deep tissue studies, may not yield additional data, thus limiting its diagnostic impact.

Elastography utilizes ultrasound images to quantify tissue deformations induced by the application of a force. Although it does not allow for a direct assessment of the mechanical properties of the tissue, this technique analyzes the tissue’s response to an external mechanical stress, translating these data into a format interpretable by the observer. Elastography enriches the information provided by traditional ultrasonography, integrating it with parameters that allow for an objective and reproducible estimation of the elastic characteristics of the examined tissues (Figure 1). In general, elastography techniques differ in the way the loading force is applied to the tissue to be examined. The quasi-static method, known as strain elastography (SE), was the first elastographic technique introduced into clinical practice. This approach involves the operator applying a static compression from the outside, allowing for the estimation of tissue deformation by comparing the initial B-mode ultrasound image (considered as a reference) with that obtained immediately after the application of the impulse. However, this technique has some limitations: (1) the magnitude of the static compression cannot be quantified, being dependent on the operator; (2) its use is limited to the study of superficial organs such as the breast and thyroid. To examine deeper organs, such as the liver and kidney, endogenous sources of mechanical stimulation can be employed, such as cardiac/arterial pulsations or respiration [6]. In dynamic methods, known as shear wave elastography (SWE), the loading force is represented by an acoustic radiation, which can be transient and of short duration or oscillatory with a fixed frequency. In both cases, the applied force generates a perturbation that deforms the tissue both in the longitudinal direction (compressional waves) and perpendicularly (shear waves). The compressional acoustic waves used in B-mode ultrasound travel through tissues at high speeds (1450–1550 m/s), while shear waves are significantly slower (1–10 m/s). SWE uses compressional acoustic waves to generate and intercept shear waves, estimating their propagation speed (shear wave speed, SWS) and algebraically calculating the Young’s modulus of the examined tissue. Dynamic elastography techniques offer the possibility of processing an elastogram with a higher resolution compared to the quasi-static method; however, the need for a transducer capable of generating shear waves, measuring the minimal tissue deformations induced, and limiting overlaps with compression waves (which can mix and become indistinguishable when passing through tissues with different acoustic impedance) requires more complex and expensive technologies [7]. The following elastographic techniques are of current interest in nephrology, and for each, a brief description of the basic technical principles is given (Table 1) [8].

**Figure 2:**
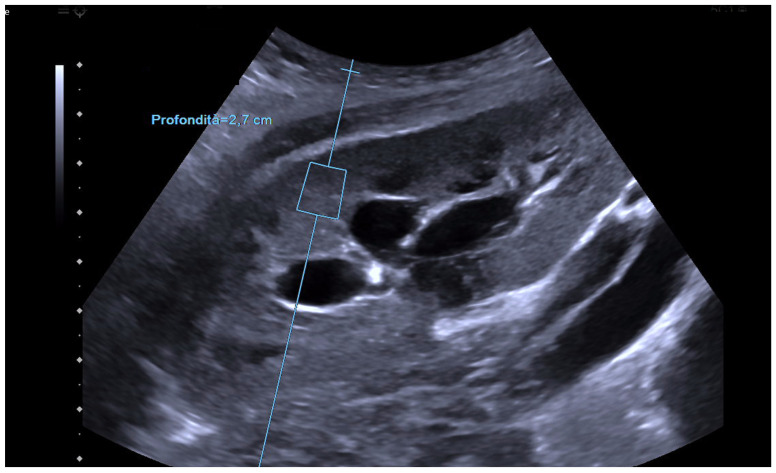
This figure illustrates the application of ARFI elastography to assess tissue stiffness in a healthy human kidney. A region of interest (ROI) was placed within the renal cortex to acquire quantitative measurements of tissue elasticity. The obtained values are consistent with the expected range for healthy adult kidney tissue.

**Figure 3:**
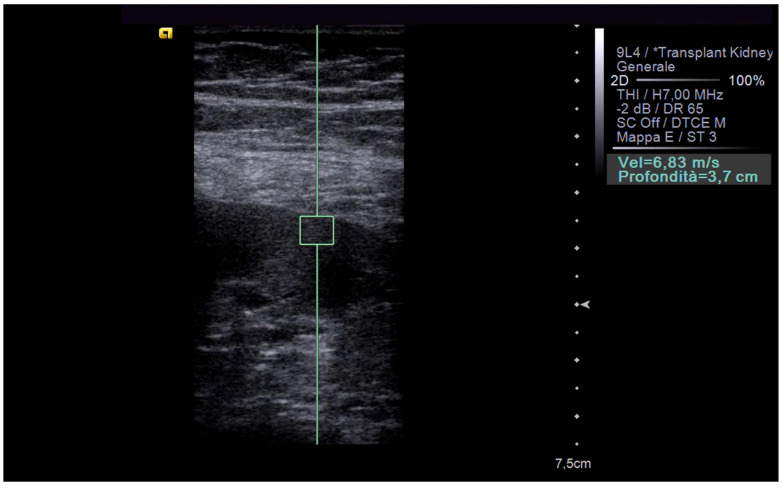
This figure presents ARFI elastography data acquired from a transplanted kidney exhibiting signs of chronic dysfunction. The region of interest (ROI) was placed within the renal cortex. Shear wave speed (SWS) measurements within the ROI were significantly elevated, approximately three times higher than the established reference range for normal transplant function. These findings align with clinical expectations for a chronically dysfunctional kidney.

Vibro-acoustography (VAG) employs two focused ultrasound beams with slightly differing frequencies to induce controlled tissue vibrations. Within the focal zone, where the beams intersect, an interference pattern emerges, causing the tissue to oscillate at the beat frequency. A sensitive hydrophone records these oscillations, distinguishing the tissue’s response frequency from the original ultrasound signals. However, VAG’s point analysis approach necessitates prolonged acquisition times, limiting its integration into routine clinical practice. Additionally, extracted data can be susceptible to artifacts stemming from variations in tissue elasticity, the magnitude of the applied force, and the geometry of the vibrating probe [9]. Acoustic Radiation Force Impulse (ARFI) uses a single focused ultrasound beam. During the emission phase, this beam generates shear waves that propagate outward from the focal point. Subsequently, the transducer alternates between emission and reception phases. In the reception phase, it analyzes the return echoes, which reflect the tissue’s response to these stresses. This analysis allows for a qualitative assessment of the stiffness (elastic properties) of the target tissue. ARFI offers several advantages: higher contrast resolution, reduced artifacts (the method minimizes signal distortions that can hinder image interpretation), less influence from the movement of surrounding tissues, and operator independence [10]. By further developing ARFI technology, Virtual Touch Tissue Quantification (VTTQ) is a dynamic elastography technique that exploits acoustic radiation force to generate and measure the propagation speed of shear waves in tissues. Unlike strain elasticity, which offers a qualitative approach, VTTQ provides a quantitative assessment, offering a numerical value for tissue stiffness (shear wave speed) as well as several advantages: quantitative data, operator independence, and better characterization of deeper tissues [10]. A variant of elastography uses a one-dimensional transient approach (1D transient elastography—1D-TE). In this methodology, the frontal surface of the transducer acts as a vibrating source, generating a low-frequency mechanical impulse that penetrates the examined tissues. This impulse creates a combination of compressional waves and spherical shear waves that propagate through the tissue. To assess tissue elasticity, a one-dimensional ultrasound transducer emits impulses and subsequently acquires backscattered echoes as a function of depth and time. This allows for the calculation of the propagation speed of the mechanical wave, which reflects the tissue stiffness [10]. It is important to note that this technique has become a mainstay in the assessment of chronic conditions such as liver fibrosis, with the FibroScan® device (Echosens, Paris, France) utilizing this patented technology [11]. A disadvantage of 1D-TE is its limited analysis area, which corresponds effectively to a sub-millimeter diameter cylinder aligned with the ultrasound transducer. Two-dimensional transient elastography—2D-TE—addresses this limitation by using a vibrating system composed of two bars positioned parallel to the ultrasound beam. These bars generate low-frequency mechanical waves that deform a significantly larger tissue area. To capture these deformations, the ultrasound transducer emits a wave simultaneously with the vibration of the bars to acquire the return echoes (Ultrafast Ultrasound Imaging—UUI). UUI technology significantly reduces the scan time per image and increases the frame rate, allowing for real-time acquisition. 2D-TE offers the advantage of examining the propagation of shear waves at multiple points simultaneously. However, the integration of the vibrating bars makes the transducer bulkier and less practical for routine clinical use [12]. Similarly to the ARFI method, shear wave imaging (SWEI) exploits mechanical stimulation and analysis of the tissue response to assess stiffness. However, there are key differences between the two. ARFI requires repeated measurements within defined areas (ROIs) due to the overlapping excitation and detection areas. In contrast, SWEI keeps the excitation beam fixed while the sensor monitors the tissue deformation along the wave propagation path, even outside the ROI. This approach allows for a quantitative assessment of the wave propagation speed (and therefore tissue stiffness), but requires complex data processing, compromising image resolution [13] (Figure 2 and Figure 3). Supersonic Shear Imaging (SSI), similar to 2D-TE, is based on acoustic radiation force rather than a vibration system to stimulate the tissue. Unlike 2D-TE, in which the focal point remains fixed, SSI uses a rapidly moving (supersonic) focal point along the longitudinal axis. This movement generates a conical wavefront, known as a Mach cone, which extends with multiple focal points, allowing for a broader assessment of the tissue under study. The transducer then briefly switches to “ultrasound interaction” mode (UUI), for less than 30 milliseconds, to capture the generated shear waves as they propagate through the tissue; this approach minimizes the impact of patient movements and allows the real-time visualization of elastograms, similar to a conventional ultrasound image. The main advantage of SSI is the use of standard ultrasound probes, eliminating the need for additional equipment and offering significant economic benefits [14]; very recently, a new patent known as Sound Touch Elastography (STE) was made commercially available. In this system, multiple shear waves are generated in different positions around an area of interest (ROI), and the system tracks the sub-millimeter displacements of the tissues and consequently derives the SWS and the corresponding Young’s modulus with reduced influence from confounding factors and improved reproducibility of the results [15].

**Table 1:** Key features and clinical applications of ultrasound elastography techniques.

Technique	Description	Reference
Vibro-acoustography	Employs two focused ultrasound beams with slightly different frequencies to induce tissue vibrations. A surface probe detects these vibrations to assess tissue stiffness on a point-by-point basis.	[9]
Acoustic Radiation Force Impulse (ARFI)	Utilizes focused ultrasound pulses to generate and measure shear waves within the tissue. The propagation speed of these waves is analyzed to determine tissue stiffness.	[10]
1D Transient Elastography (1D-TE)	Employs a single transducer to generate low-frequency mechanical waves that propagate through the tissue. Tissue stiffness is assessed by analyzing the propagation speed of these waves.	[11]
2D Transient Elastography (2D-TE)	Extends the 1D-TE technique by using multiple vibrating sources to generate shear waves across a larger area, enabling the simultaneous analysis of multiple tissue points.	[12]
Shear Wave Elastography (SWEI)	Utilizes a fixed excitation beam and a moving sensor to monitor tissue deformation along the wave propagation path, providing a quantitative assessment of tissue stiffness.	[13]
Supersonic Shear Imaging (SSI)	Employs a rapidly moving focal point to generate shear waves, enabling real-time visualization of tissue stiffness.	[14]

## 3. Elastography in the Kidney: Basic Technique and Limiting Factors

Renal stiffness assessment has historically relied on Shear Wave Elastography (SWE). This method, similar to conventional ultrasound, employs a variable-frequency convex probe (2–5 MHz), examining the kidney from various angles (anterior, posterior, lateral) and planes (longitudinal, coronal, transverse, oblique). Although no standardized protocol exists, clinical practice generally involves three to twelve elastographic measurements at various cortical and medullary points, with the average of these measurements reflecting overall renal stiffness. The examination procedure is divided into several phases. First, a conventional ultrasound evaluation is performed, providing initial information on the renal structure. Subsequently, a targeted tissue deformation is induced through a high-intensity focused pulse (acoustic radiation force), which causes a local deformation in a small region of interest (ROI) selected in the B-mode image; the choice of ROI position is aimed at reducing the influence of surrounding tissues. Thirdly, an analysis of shear waves is performed using multiple focused reading pulses, which measure the propagation speed of the generated tissue deformation waves, thus providing quantitative data on stiffness. Finally, a grayscale image allows for a qualitative assessment of tissue elasticity, proving particularly useful for high spatial resolution requirements, such as the characterization of solid lesions (Figure 2 and Figure 3) [16]. However, several factors can influence the results of SWE. One of these is renal anisotropy, a characteristic that determines how elasticity measurements, as well as the propagation speed of mechanical waves, can vary depending on the measurement technique and the direction of insonation. This phenomenon is linked to the structural organization of the kidney, with faster wave propagation when parallel to tubular structures and arteries, compared to perpendicular measurements [17]. Another aspect to consider is viscoelasticity; the generation of shear waves tends to decrease in tissues with a high fluid content, underlining the importance of positioning the ROI away from vessels and cysts. Blood perfusion also plays a significant role—alterations such as glomerulosclerosis, and vascular problems such as renal artery stenosis or venous thrombosis, can significantly influence shear wave speed (SWS), with reported variations exceeding 500%; moreover, hydronephrosis can cause substantial changes, up to 137% [18,19]. A further critical aspect is the depth of the ROI—as in other elastographic techniques, depth affects measurements. A study conducted by Bota et al. showed that, in the same tissue sample, SWS can decrease by up to 27% as the ROI depth increases by 3 cm [20]. This represents a particular challenge for obese patients or those with abnormalities in kidney position, potentially compromising the reliability of elastography. Although reference values for shear wave speed (SWS) seem applicable to the ARFI technique, their validity for other elastographic methodologies remains uncertain. Studies have shown significant discrepancies in SWS measurements between healthy adults when different elastographic techniques are used [19]. The study by Singh et al. examined shear wave speed (SWS) in various abdominal organs of healthy subjects, revealing that kidneys show the highest variability in elasticity values compared to other organs using the ARFI technique [20]. Table 2 shows the normal reference values of SWS proposed by some authors for the cortical region of native and transplanted kidneys. For native kidneys with normal function in young and adult patients, the mean SWS, based on ARFI measurements, varies between 2.15 and 2.54 m/s [15,16,17,18,19,20], corresponding to an average stiffness of 15.4 ± 2.5 kPa for the renal cortex [15,18]. Furthermore, it is interesting to note that SWS changes with age. Healthy children have the highest values (mean SWS ± standard deviation: 3.13 ± 0.09 m/s), while the elderly show a significant decrease, even with preserved renal function (mean ± standard deviation: 1.82 ± 0.63 m/s) [14,19].

**Table 2:** This table presents a summary of some recent studies utilizing elastography to measure shear wave speed (SWS) or Young modulus in both native and transplanted kidneys with normal function. The values reported represent the mean SWS measurements obtained from the cortical parenchyma of the kidneys.

	Shear Wave Speed (m/s, Mean and SD) or Young Modules	Authors	Reference
	1.82 ± 0.63	Bota et al.	[19]
**Native Kidney**	1.49 ± 0.19 (right kidney)	Singh et al.	[20]
	1.54 ± 0.19 (left kidney)		
**Transplant Kidney**	<30.95 kPa	Yang et al.	[15]
	<2.625 m/s	He et al.	[21]
	<2.83 m/s	Liu et al.	[22]

## 4. Elastography in Chronic Dysfunction of the Transplanted Kidney: State of the Art

While the depth of the tissue to be examined represents the main limitation of elastography, the transplanted kidney, thanks to its superficial position, theoretically lends itself well to sampling for the non-invasive assessment of tissue stiffness. In the last fifteen years, numerous original studies have explored the role of elastography in kidney transplantation, particularly for the evaluation of chronic dysfunction. Initial results, based on pioneering studies with very limited samples, were often conflicting: more recent research shows more promising outcomes. The first study, conducted by Stock et al., identified a moderate association between SWE and the degree of fibrosis in a group of 18 transplanted patients, but found no correlation between SWS and parenchymal resistance indices [23]. In contrast, Syersveen et al., in their case series involving 31 transplanted patients with biopsy-confirmed fibrosis, found no significant correlation between SWE values and fibrosis grading [24]. Ren et al., in a study of 74 transplanted patients, discovered that SWE values measured in the renal cortex were significantly higher in patients with renal dysfunction and high intraparenchymal resistance indices compared to those with normal renal function; this same study also found that SWS values were significantly higher in the cortex compared to the medullary region and renal sinus [25]. In support of ARFI’s potential, He et al. conducted the largest case series reported, involving 102 transplanted patients. They concluded that SWE, with a threshold value of 2.625 m/s, provided greater accuracy than resistance indices in the diagnosis of post-transplant renal dysfunction, also highlighting an inverse correlation between SWS and GFR [21]. In 2013, Ozkan et al. described that, in a sample of 42 patients evaluated with RT elastosonography, there was a significant correlation between parenchymal stiffness assessed in terms of Young’s modulus and intraparenchymal RIs (r = 0.41; *p* = 0.007), but also demonstrated that the variability in measurements between operators was particularly wide (interclass correlation 0.47) [26]. A year later, Lukenda et al. published an original article on a sample of 52 kidney transplant patients, in which parenchymal stiffness was studied with transient elastography (TE), finding an inverse correlation between SWE and eGFR values (r = −0.64; *p* < 0.0001) and a positive correlation between SWE and the fibrosis grading found on biopsy (r = 0.727; *p* = 0.0001) [27]. Dai et al., in their study of 54 kidney transplant patients, used the ARFI-VTTQ technique, finding a significant correlation between tissue stiffness, measured using this algorithm, and the degree of parenchymal fibrosis [28]. Liu et al. reported a diagnostic accuracy of 78.7% in predicting chronic organ dysfunction with an SWS threshold value of 2.83 m/s in a cohort of 28 transplanted patients [22]. In 2020, Gokalp et al. evaluated the relationship between SWV changes and biopsy outcome in a prospective study of 34 patients with end-stage renal failure; the comparison was made by evaluating SWV pre-transplant, at the time of implantation, and six months after implantation, and a positive correlation was found between the histological grading of inflammatory infiltrate and tubulitis in patients with rejection and the variation in SWE, while no correlation was found between the variation in SWV and the peritubular capillaritis score. The authors concluded that the change in SWE can predict an acute reaction in kidney transplant recipients [29]. Bolboaca et al. in 2020 published an original study centered on the variability in SWE measurements. In particular, this study analyzed data on 83 patients from a single center, 69 with stable renal function and 14 with suspected organ dysfunction (assessed as an increase in creatinine >0.3 mg/dL or >50% compared to baseline or proteinuria >1 gr/day). The elastography study was performed using a high-frequency linear probe, targeting the cortical and medullary regions of the renal graft. This approach differs from many similar studies, which typically employ a convex probe. Importantly, these authors determined that there was no significant difference in vascularization, assessed as intraparenchymal RIs, between patients with organ dysfunction and patients with normal function. This study confirmed that patients with renal dysfunction have significantly higher stiffness compared to patients with normal graft function (median 28.70 kPa vs. 20.99 kPa, *p* value *p* = 0.0142); a novel element introduced by this article is that both cortical and medullary stiffness correlate positively with the proteinuria/creatinuria ratio (r = 0.33; *p* = 0.0021). However, this study also demonstrated that intra-examination measurements (same operator for the same organ examined) present significant heterogeneity, especially in the group of patients with organ dysfunction, up to 42.38% [30]. Chhhajer et al. in 2021 published an original cross-sectional study in which the outcomes of 172 kidney biopsies graded with the semi-quantitative Banff score were compared with the average of three SWE measurements (acquired with an ROI of at least 1 cm^2^ on three different renal segments) and with the average of three intraparenchymal RI measurements. The authors found that there was a significant correlation between the Banff grade and the SWE score assigned to the patient with a correlation coefficient of 0.665 (*p* < 0.001); creatinine values at the time of biopsy also correlated with SWE and Banff grading. Differently from other studies, no significant correlation was found between the histological grading and RIs, nor was any correspondence found with patient age. Importantly, a value of 4.4 kPa was indicated as a cut-off to differentiate low-grade fibrosis (Banff 0–1) from high-grade fibrosis (Banff 2–3) with a positive predictive value of 71% and a negative predictive value of 93.8%. In the Chhhajer et al. study, evaluations were performed on three different segments of the kidney (upper, middle, and lower pole), and it was found that there is a greater significance between the degree of SWE at the lower pole and the Banff grading found. In the context of the study reported, the biopsies were all performed on the lower pole, but it is not clear whether this depends on a different distribution of fibrosis or, more likely, on a more punctual estimate at that level. In any case, a targeted assessment of the lower pole could be useful to standardize the methodology [31]. Barsoum et al., on a rather limited sample (36 patients), demonstrated that SWE values had a statistically significant positive correlation with the time elapsed since transplantation, as well as with the Banff score, defining 28.64 kPa as a cut-off value with a sensitivity of 87% and specificity of 90% for severe fibrosis [32]. Eisingergy et al. in 2023 published an original study comparing ultrasound elastosonography and MRI elastography in a small cohort of pediatric and adolescent patients that included 18 transplanted patients and 8 healthy controls. The authors demonstrated that the average ultrasonographic stiffness measured was significantly higher in patients with graft dysfunction (mean 23.4 kPa), compared to those with stable function (mean 13.7 kPa) and controls (mean 9.1 kPa); they also described a positive correlation between SWE values and the extent of organ fibrosis, and that a cut-off value of 13.8 kPa results in a specificity of 72% and a sensitivity of 100% in detecting organ fibrosis on biopsy. This pilot study (although limited by the small sample size) is currently the most recent original article reported in the literature for the assessment of organ dysfunction in the transplanted kidney in non-adult age [33]. In the context of the applications of elastography to transplantation, only a few studies in the literature have prospectively analyzed the prognostic value of SWE. Among these, the work of Zhang et al., published in 2023, is to our knowledge the most recent and also that with the largest population. This single-center prospective study included a total of 161 kidney transplant patients, with evaluation of the following primary endpoints: worsening renal function with loss >25% of eGFR, rehospitalization for all causes, all-cause mortality. In patients who reached one of the primary endpoints, it was found that intraparenchymal RIs were significantly higher (mean 0.69 vs. 0.66; *p* = 0.030) and had greater stiffness of the medullary regions (mean SWE 10.46 vs. 9.55; *p* = 0.022). Interestingly, the analysis of ROC curves and Kaplan–Meier curves produced by the authors showed that the median stiffness of the medullary regions has the best predictive accuracy for primary outcomes when compared to cortical stiffness and RIs, and that this is the most important discriminating factor to define outcomes in the sample [34]. Yang et al. further investigated the use of elastography for the evaluation of kidney transplant patients with suspected CAN based on laboratory findings (creatinine values, eGFR < mL/min/1.73 m^2^); in this very recent original cross-sectional study, the authors evaluated, among the ultrasound parameters, the intraparenchymal RIs calculated on the arcuate artery and two different types of elastography on the cortical regions: STE and SE. The authors demonstrated that Young’s modulus values have a strong negative correlation with eGFR (r = 0.713; *p* < 0.001), while RIs and the renal function are only moderately inversely correlated; importantly, in this paper, it is defined that a Young’s modulus value of 30.95 kPa can be considered indicative of CAN with a sensitivity of 92%, a specificity of 88%, and an accuracy of 88%; [15]. In 2024, Jesrani et al. published a single-center cross-sectional study involving 154 patients undergoing kidney transplant biopsy at least three months after transplantation and who had shown an increase >20% in creatinine values or a reduction of <50 mL/min of creatinine clearance; the patients were studied ultrasonographically with SWE and intraparenchymal RI assessment, and they subsequently underwent biopsy at the segment with the highest SWE values; semi-quantitative biopsy scores were used in the final analysis to obtain the diagnostic accuracy of SWE and RI. The authors described that in relation to the ability to detect chronic changes in the allograft, sensitivity, specificity, positive predictive value, negative predictive value, and diagnostic accuracy were, respectively, 93.10%, 96.87%, 97.73%, 95.87%, and 95.45% for SWE, while significantly lower performance was recognized in the assessment of Ris, with results of, respectively, 76.92%, 83.33%, 70.17%, 87.62%, and 81.16% for the same considered items [35]. These studies are summarized in Table 3.

**Table 3:** Summary of key studies on renal elastography and chronic renal transplant dysfunction (CAN).

Authors	Reference	Year	Patient Number	Elastographic Assessment	Region Explored	Bioptic Assessment	Main Conclusions
Stock et al.	[23]	2011	18	Yes	Cortical	Yes	Moderate correlation between SWS and degree of fibrosis, no correlation with intraparenchymal IR
Syersveen et al.	[24]	2012	31	Yes	-	Yes	No correlation between SWS and fibrosis grading
Ren et al.	[25]	2013	74	Yes	Cortical, medullary, renal sinus	No	Significant correlation between intraparenchymal SER and IR vs. renal function indices
He et al.	[21]	2013	102	Yes	-	No	Inverse correlation between SWE and eGFR, SWE > 2.625 m/s proposed as a cutoff value to define chronic dysfunction
Ozkan et al.	[26]	2013	42	Yes	-	No	Significativa correlazione tra kPa rilevata e IR, elevata variabilità inter-osservatore
Lukenda et al.	[27]	2014	52	Yes	-	No	Significant correlation between detected kPa and IR, high inter-observer variability
Dai et al.	[28]	2014	54	Yes	-	Yes	Significant correlation between tissue stiffness assessed with ARFI and fibrosis grading in biopsy
Liu et al.	[22]	2013	28	Yes	-	No	SWE > 2.83 m/s results in a diagnostic accuracy of 78.7% in predicting chronic dysfunction
Gokalp et al.	[29]	2020	34	Yes	-	Yes	Positive correlation between SWE variation and inflammatory infiltrate
Bolboaca et al.	[30]	2020	83	Yes	Cortical, medullary	No	Positive correlation between stiffness of cortical and medullary regions and proteinuria/creatinuria ratio, significant variability in intra-operator observations
Chhajer et al.	[31]	2021	172	Yes	Upper, middle, lower pole	Yes	Significant correlation between SWE and Banff grade, no correlation between IR and Banff score
Barsoum et al.	[32]	2022	36	Yes	-	Yes	Positive correlation between SWE and time since transplant, and between SWE and Banff score
Eisingergy et al.	[33]	2023	10	Yes	-	Yes	Positive correlation between SWE and biochemical signs of organ dysfunction and between SWE and Banff score in patients undergoing biopsy
Zhang et al.	[34]	2023	161	Yes	Cortical, medullary	No	Renal medullary region stiffness predictive of primary study outcome (>25% reduction in eGFR or all-cause mortality)
Yang et al.	[15]	2023	101	Yes	Cortical	No	Young’s modulus strongly correlates with decreased eGFR, while IR shows a weaker negative correlation. A 30.95% cut-off value accurately diagnoses CAN (biochemically suspected) with high sensitivity and specificity
Jesrani et al.	[35]	2024	154	Yes	-	Yes	High diagnostic accuracy of SWE for chronic changes

## 5. Considerations and Conclusions

Over the past two decades, there has been a growing interest among researchers in the application of parametric techniques to the study of the transplanted kidney. Starting from the first pioneering studies over fifteen years ago and especially in the last five years, there has been a veritable explosion of publications. While the first prospective and cross-sectional studies were based on numerically small samples (unlike more recent research), and the initial results were sometimes contradictory, it can now be stated that elastometry parameters are almost invariably correlated with histological grading, considered the gold standard for the diagnosis of organ fibrosis. Most studies, in fact, have shown a positive correlation between SWE values and the degree of renal fibrosis, confirming the hypothesis that the increase in tissue stiffness reflects an already ongoing fibrotic process. This result confirms the rational expectations for the use of elastometry and represents the main contribution of this technique to the study of the transplanted kidney. Its non-invasiveness, low cost, and the possibility of performing routine measurements even in peripheral centers justify its large-scale adoption. An interesting, and still controversial, aspect is that intraparenchymal resistance indices (IR), traditionally considered among the most relevant parameters in ultrasound nephrological diagnostics, do not always show a correlation with elastometric parameters, except in cases of more advanced fibrosis. This observation, which emerges from the literature, suggests that parenchymal fibrosis may precede the development of nephroangiosclerosis in the transplanted kidney, although this hypothesis requires further confirmation from experimental studies on animal models; in any case, SWE seems to offer greater diagnostic accuracy than resistance indices (IR) in the evaluation of renal fibrosis, suggesting a potential role as a non-invasive biomarker. The prognostic value of elastography has also been explored, indicating that tissue stiffness could be an independent predictor of adverse events such as worsening renal function and rehospitalization. A further relevant element is the intra- and inter-observer variability, highlighted by all the studies that have analyzed this aspect. This high variability represents the main limitation of elastometry, as discrepancies between successive measurements can lead to erroneous diagnostic evaluations, with potential repercussions on the therapeutic level, and moreover, the variability in measurements, in particular between different operators, representing a significant limitation that could influence the reproducibility of the results. Despite the fact that several studies have proposed possible cut-off levels for the detection of organ fibrosis, to date there is no unique and shared criterion; this fact, together with the lack of standardized protocols for the acquisition and analysis of elastographic data and the considerable heterogeneity of the samples in terms of numbers and also type of patient, limits the comparability between the different studies. Consequently, if the diagnosis of fibrosis were decisive for a therapeutic choice, it is not yet recommended to abandon invasive diagnostics in favor of elastography. It is hoped that multicenter studies will be conducted to establish, with a higher level of evidence, definitive parameters such as SWE or Young’s modulus for the non-invasive diagnosis of fibrosis. However, significant variations in the mean SWE values between two different follow-up measurements can already indicate, with a sufficient degree of confidence, a negative prognostic factor for chronic graft dysfunction, suggesting the need for adjustments in subsequent follow-up. Elastography appears to be a promising non-invasive technique for the evaluation of renal fibrosis in transplanted patients. However, further studies are needed to clarify its role in clinical practice and to optimize the procedures for acquiring and analyzing data. The standardization of protocols and the development of more accurate predictive models represent the next challenges in this field of research.
